# Quality of life in ATTR amyloidosis

**DOI:** 10.1186/1750-1172-10-S1-O26

**Published:** 2015-11-02

**Authors:** Thirusha Lane, Alica Bangova, Marianna Fontana, David F Hutt, Svetla G Strehina, Carol J Whelan, Philip N Hawkins, Julian D Gillmore

**Affiliations:** 1 University College London, National Amyloidosis Centre, NW3 2PF, London, UK

## Background

Quality of life (QoL) is of paramount importance in chronic diseases. There is anecdotal evidence on the poor QoL of patients with ATTR amyloidosis, but few or no prospective data. In an era of development of disease-modifying drugs, it has become extremely important to quantify their effects on QoL.

## Methods

As part of a protocolised model of care involving comprehensive annual clinical evaluation (including DPD scintigraphy, echocardiography, ECG, neurological testing, cardiac magnetic resonance imaging (CMR), 6-minute walk test and blood tests), patients completed the KCCQ and SF-36 questionnaires. The KCCQ quantifies physical function (PF), symptoms, social function (SF), and QoL in cardiomyopathy. The SF-36 is a general health survey which measures functional health and well-being. Individual domains in both questionnaires are scored out of a maximum of 100, with scores closer to 100 representing lower disease burden.

## Results

Over a 2½ year period, 179 KCCQs were completed by 137 patients – 74 wild-type (WT), 22 V122I, 21 T60A, 4 V30M and 10 with various other mutations. 5 patients were asymptomatic V30M carriers and 1 was a domino transplant recipient, also asymptomatic. Patients with a phenotype of cardiac-isolated disease (WT and V122I) showed significant limitation of PF (scores out of 100, 54 and 46 respectively) and SF (58 and 32), greater symptom burden (64 and 50) and poorer overall QoL (59 and 41). 38 patients repeated the questionnaire over a one or two year period, WT patients making up 25 of the 38. 60% of WT patients showed significant worsening in PF, SF and QoL over the 1-2 year follow-up period. Over the same time period, 167 SF-36 health surveys were completed by 132 patients, including 33 who repeated the assessment at least annually. Impairment of PF and SF and general health (GH) in the cardiac-isolated cohort were comparable with that indicated by the KCCQ, with the V122I patients scoring worse than the WT group, and with similar deterioration over the follow-up period. However, the SF-36 revealed poor scores in nearly all domains in patients with a neuropathy-dominant phenotype, in some domains comparable with that of the V122I group - PF scores: WT 39, V122I 34, neuropathy-dominant 34; SF scores: 65, 53, 55; Bodily pain: 61, 49, 51; GH: 45, 35, 37; Vitality: 43, 41, 44 respectively. Grade of DPD uptake, extracellular volume on CMR, and 6-minute walk test distance all reflected physical disease burden as measured by both questionnaires.

## Conclusion

Patients with ATTR amyloidosis have significant impairment over several areas of health and holistic well-being, resulting in generally poor QoL. Those with the V122I variant appeared to show the greatest disease burden in all domains, but patients with neuropathic phenotypes also exhibited severe limitation. QoL deteriorated markedly over 1-2 years follow-up. The effect of the various new treatments in development is eagerly awaited.

